# Supplementary material for: 3′-UTR Polymorphisms in Thymidylate Synthase with Colorectal Cancer Prevalence and Prognosis
Source: J Pers Med. 2021 Jun 9;11(6):537. doi: 10.3390/jpm11060537 (PMC8228787; doi:10.3390/jpm11060537)
Supplement: Supplementary file 1 [file jpm-11-00537-s001.zip › jpm-1218599-supplementary.pdf]

Table S1

Adjusted odds ratios of gene-environment combined effects on CRC incidence.

	HTN		DM		HDL-C		Hcy		FA	
Genotypes	No	Yes	No	Yes	≥40(M)/50(F)	<40(M)/50(F)	<11.7	≥11.7	≥4.58	<4.58
1100TT	1.00 (ref)	2.65 (1.70 - 4.12)	1.00 (ref)	1.13 (0.74 - 1.72)	1.00 (ref)	3.11 (1.96 - 4.92)	1.00 (ref)	0.84 (0.50 - 1.41)	1.00 (ref)	2.14 (1.25 - 3.66)
1100TC+CC	0.97 (0.64 - 1.47)	2.58 (1.66 - 4.00)	0.85 (0.57 - 1.28)	1.38 (0.91 - 2.09)	0.98 (0.69 - 1.38)	3.43 (2.13 - 5.53)	0.89 (0.63 - 1.27)	1.06 (0.63 - 1.78)	0.97 (0.68 - 1.38)	2.21 (1.34 - 3.63)
1170AA	1.00 (ref)	2.15 (1.31 - 3.52)	1.00 (ref)	1.67 (1.05 - 2.67)	1.00 (ref)	2.70 (1.65 - 4.42)	1.00 (ref)	1.26 (0.72 - 2.22)	1.00 (ref)	2.51 (1.45 - 4.33)
1170AG+GG	1.81 (1.19 - 2.75)	4.19 (2.65 - 6.62)	2.14 (1.40 - 3.27)	2.15 (1.38 - 3.36)	1.71 (1.20 - 2.43)	5.27 (3.30 - 8.42)	1.84 (1.28 - 2.63)	1.72 (1.03 - 2.86)	1.83 (1.27 - 2.64)	4.04 (2.39 - 6.83)
1100TT-1170AA	1.00 (ref)	1.39 (0.51 - 3.78)	1.00 (ref)	2.54 (0.99 - 6.52)	1.00 (ref)	3.72 (1.45 - 9.51)	1.00 (ref)	1.55 (0.50 - 5.28)	1.00 (ref)	3.55 (1.12 - 11.27)
1100TT-1170AG	1.40 (0.66 - 3.00)	4.07 (1.89 - 8.78)	3.29 (1.38 - 7.84)	2.48 (1.05 - 5.84)	2.12 (1.07 - 4.20)	6.83 (3.06 - 15.24)	2.11 (1.10 - 4.07)	1.90 (0.77 - 4.69)	2.00 (1.02 - 3.92)	5.50 (2.27 - 13.31)
1100TT-1170GG	4.88 (1.87 - 12.78)	7.40 (2.74 - 19.99)	5.93 (2.27 - 15.50)	10.77 (3.49 - 33.20)	7.69 (3.21 - 18.44)	10.29 (3.59 - 29.45)	5.45 (2.40 - 12.35)	5.10 (1.50 - 17.37)	8.36 (3.48 - 20.08)	8.54 (2.25 - 32.44)
1100TC-1170AA	1.13 (0.53 - 2.44)	2.61 (1.17 - 5.84)	2.44 (0.96 - 6.20)	2.63 (1.12 - 6.17)	1.64 (0.82 - 3.29)	4.74 (2.07 - 10.84)	1.41 (0.73 - 2.72)	1.99 (0.79 - 5.00)	1.53 (0.77 - 3.04)	3.33 (1.38 - 8.05)
1100TC-1170AG	2.60 (1.17 - 5.79)	5.84 (2.35 - 14.52)	3.56 (1.43 - 8.86)	6.77 (2.56 - 17.85)	3.02 (1.48 - 6.16)	13.92 (5.28 - 36.70)	2.88 (1.40 - 5.96)	3.21 (1.14 - 9.00)	3.43 (1.62 - 7.27)	7.56 (2.83 - 20.19)
1100CC-1170AA	2.07 (0.73 - 5.85)	5.19 (1.73 - 15.55)	2.09 (0.74 - 5.95)	11.23 (3.23 - 39.10)	3.60 (1.50 - 8.66)	8.29 (2.18 - 31.54)	3.14 (1.25 - 7.89)	4.41 (1.02 - 19.03)	3.84 (1.43 - 10.35)	10.86 (2.60 - 45.42)
TA haplotype	1.00 (ref)	2.58 (1.78 - 3.76)	1.00 (ref)	1.20 (0.84 - 1.71)	1.00 (ref)	3.49 (2.40 - 5.09)	1.00 (ref)	1.06 (0.68 - 1.65)	1.00 (ref)	2.48 (1.62 - 3.81)
TG haplotype	1.99 (1.40 - 2.83)	5.15 (3.51 - 7.57)	2.04 (1.44 - 2.90)	2.27 (1.56 - 3.31)	2.09 (1.55 - 2.81)	6.22 (4.14 - 9.36)	1.97 (1.46 - 2.66)	1.80 (1.16 - 2.82)	2.12 (1.57 - 2.86)	4.25 (2.67 - 6.75)
CA haplotype	1.43 (0.99 - 2.07)	3.79 (2.55 - 5.63)	1.27 (0.87 - 1.84)	2.16 (1.47 - 3.17)	1.56 (1.15 - 2.12)	4.77 (3.09 - 7.36)	1.35 (0.99 - 1.83)	1.80 (1.11 - 2.91)	1.50 (1.09 - 2.06)	3.67 (2.32 - 5.81)

HTN, hypertension; DM, diabetes mellitus; HDL-C, high density lipoprotein-cholesterol; M, male; F, female; Hcy, homocysteine; FA, folate. Haplotypes of frequencies<5% were excluded from the analysis.

The units of HDL-C, Hcy, and FA were mg/dl, µmol/l, and ng/ml, respectively.

11.7 was upper 25% cut-off value of Hcy in total participants.

4.58 was lower 25% cut-off value of FA in total participants.

Odds ratios were adjusted by age, gender, HTN, DM, BMI, and HDL-C.

Table S2

Survival analysis of *TS* 3'-UTR polymorphisms in CRC patients

		Overall survival			Relapse-free survival		
Genotypes	Patients (n=350)	Death (n=61)	AHR (95% CI)	*P*	Relapse (n=64)	AHR (95% CI)	*P*
TS 1100T>C							
TT	180 (51.4)	26 (42.6)	1.00 (ref)		27 (42.2)	1.00 (ref)	
TC	141 (40.3)	26 (42.6)	1.90 (1.08 - 3.36)	0.027	28 (43.8)	2.13 (1.22 - 3.73)	0.008
CC	29 (8.3)	9 (14.8)	2.37 (1.02 - 5.49)	0.046	9 (14.1)	2.26 (0.95 - 5.40)	0.068
Dominant			1.82 (1.07 - 3.08)	0.027		2.00 (1.19 - 3.35)	0.009
Recessive			1.36 (0.63 - 2.93)	0.434		1.30 (0.60 - 2.83)	0.515
TS 1170A>G							
AA	126 (36.0)	27 (44.3)	1.00 (reference)		28 (43.8)	1.00 (reference)	
AG	156 (44.6)	23 (37.7)	0.82 (0.46 - 1.48)	0.516	27 (42.2)	0.94 (0.54 - 1.65)	0.836
GG	68 (19.4)	11 (18.0)	0.63 (0.30 - 1.31)	0.217	9 (14.1)	0.45 (0.20 - 0.98)	0.046
Dominant			0.77 (0.45 - 1.30)	0.328		0.74 (0.44 - 1.24)	0.253
Recessive			0.70 (0.35 - 1.38)	0.301		0.48 (0.23 - 1.00)	0.050

Abbreviation: AHR: adjusted hazard ratio.

Adjusted by age, gender, tumor size, tumor site, tumor differentiation, TNM stage, and chemotherapy.

Table S3

Survival analysis for combined genotypes and haplotypes *TS* in CRC patients

Characteristics		Patients (n=350)	Overall survival			Relapse-free survival		
			Death (n=61)	AHR (95% CI)	*P*	Relapse (n=64)	AHR (95% CI)	*P*
TS 1100	*TS* 1170							
TT	AA	26 (7.4)	4 (6.6)	1.00 (ref)		5 (7.8)	1.00 (ref)	
TT	AG	86 (24.6)	11 (18.0)	1.01 (0.31 - 3.32)	0.993	13 (20.3)	0.91 (0.32 - 2.59)	0.856
TT	GG	68 (19.4)	11 (18.0)	1.22 (0.36 - 4.07)	0.751	9 (14.1)	0.83 (0.26 - 2.72)	0.763
TC	AA	71 (20.3)	14 (23.0)	1.35 (0.43 - 4.24)	0.611	14 (21.9)	1.09 (0.39 - 3.06)	0.877
TC	AG	70 (20.0)	12 (19.7)	1.47 (0.45 - 4.80)	0.529	14 (21.9)	1.17 (0.41 - 3.33)	0.770
CC	AA	29 (8.3)	9 (14.8)	2.62 (0.66 - 10.43)	0.175	9 (14.1)	2.02 (0.58 - 7.05)	0.275
TS haplotype								
1100T-1170A		209 (29.9)	33 (27.0)	1.00 (ref)		37 (28.9)	1.00 (ref)	
1100T-1170G		292 (41.7)	45 (36.9)	0.98 (0.60 - 1.59)	0.930	45 (35.2)	0.83 (0.52 - 1.32)	0.426
1100C-1170A		199 (28.4)	44 (36.1)	1.51 (0.94 - 2.44)	0.089	46 (35.9)	1.38 (0.87 - 2.19)	0.168

Abbreviation: AHR: adjusted hazard ratio.

Haplotypes of frequencies<5% were excluded from the analysis.

Adjusted by age, gender, tumor size, tumor site, tumor differentiation, TNM stage, and chemotherapy.

Table S4

Adjusted hazard ratios of prognosis risk factors on CRC patients according to TS 3'-UTR polymorphisms

	**TNM stage**		**Differentiation**		**HDL-C<40(male)/50(female)mg/dl**		**Hcy≥12.4μmol/l**		**FA<3.94ng/ml**	
Characteristics	OS	RFS	OS	RFS	OS	RFS	OS	RFS	OS	RFS
1100TT	4.20 (2.41 - 7.34)	5.25 (3.00 - 9.22)	3.05 (1.22 - 7.61)	2.58 (1.01 - 6.61)	2.69 (1.17 - 6.16)	1.79 (0.83 - 3.84)	1.44 (0.63 - 3.31)	1.39 (0.61 - 3.17)	1.89 (0.86 - 4.14)	1.49 (0.67 - 3.31)
1100TC+CC	4.63 (2.70 - 7.95)	7.03 (4.02 - 12.27)	3.62 (1.53 - 8.58)	2.91 (1.21 - 6.98)	1.60 (0.81 - 3.13)	1.67 (0.87 - 3.20)	2.21 (1.14 - 4.31)	1.67 (0.85 - 3.27)	2.23 (1.13 - 4.41)	2.31 (1.19 - 4.49)
1170AA	3.80 (2.10 - 6.90)	6.19 (3.39 - 11.31)	3.67 (1.54 - 8.75)	2.86 (1.18 - 6.98)	0.90 (0.42 - 1.90)	0.88 (0.42 - 1.84)	2.30 (1.07 - 4.95)	1.67 (0.76 - 3.67)	2.40 (1.11 - 5.19)	2.03 (0.95 - 4.31)
1170AG+GG	4.54 (2.77 - 7.45)	5.28 (3.23 - 8.62)	2.71 (1.11 - 6.64)	2.37 (0.96 - 5.87)	3.67 (1.72 - 7.82)	2.78 (1.39 - 5.54)	1.62 (0.81 - 3.27)	1.54 (0.77 - 3.07)	1.84 (0.91 - 3.71)	1.70 (0.85 - 3.39)
1100TT-1170AA	2.56 (0.60 - 10.98)	3.29 (0.90 - 12.09)	2.55 (0.31 - 21.31)	2.43 (0.38 - 15.69)	1.57 (0.16 - 15.10)	0.88 (0.15 - 5.24)	2.45 (0.25 - 23.59)	1.55 (0.17 - 13.76)	1.06 (0.10 - 10.73)	0.46 (0.05 - 4.06)
1100TT-1170AG	5.85 (2.16 - 15.84)	8.36 (3.28 - 21.31)	3.58 (0.96 - 13.42)	3.60 (1.02 - 12.74)	2.61 (0.70 - 9.79)	1.71 (0.56 - 5.21)	0.91 (0.24 - 3.42)	1.67 (0.55 - 5.09)	2.35 (0.72 - 7.66)	1.73 (0.58 - 5.12)
1100TT-1170GG	3.85 (1.71 - 8.68)	4.47 (1.79 - 11.16)	2.85 (0.49 - 16.64)	1.12 (0.20 - 6.19)	3.22 (0.95 - 10.95)	2.37 (0.64 - 8.80)	2.11 (0.62 - 7.16)	1.07 (0.22 - 5.09)	2.67 (0.71 - 10.01)	2.02 (0.42 - 9.65)
1100TC-1170AA	5.16 (1.91 - 13.91)	8.02 (2.95 - 21.81)	5.39 (1.36 - 21.29)	2.83 (0.59 - 13.62)	1.11 (0.39 - 3.18)	1.16 (0.41 - 3.33)	3.15 (1.11 - 8.92)	2.68 (0.93 - 7.68)	4.02 (1.42 - 11.43)	3.97 (1.39 - 11.28)
1100TC-1170AG	5.68 (2.26 - 14.28)	6.39 (2.51 - 16.22)	2.11 (0.32 - 13.64)	2.47 (0.44 - 13.74)	6.66 (1.47 - 30.19)	5.36 (1.50 - 19.13)	2.14 (0.68 - 6.70)	1.74 (0.59 - 5.17)	1.30 (0.35 - 4.78)	1.57 (0.50 - 5.00)
1100CC-1170AA	3.44 (1.25 - 9.43)	7.25 (2.32 - 22.62)	2.20 (0.58 - 8.35)	2.40 (0.63 - 9.11)	0.62 (0.16 - 2.49)	0.61 (0.15 - 2.47)	1.19 (0.30 - 4.73)	0.59 (0.12 - 2.81)	1.63 (0.41 - 6.51)	1.37 (0.34 - 5.43)
TA haplotype	4.25 (2.47 - 7.32)	5.96 (3.53 - 10.05)	3.77 (1.72 - 8.25)	3.01 (1.38 - 6.53)	1.63 (0.79 - 3.36)	1.28 (0.67 - 2.47)	1.03 (0.96 - 1.10)	1.04 (0.97 - 1.11)	0.94 (0.87 - 1.03)	0.95 (0.88 - 1.03)
TG haplotype	4.27 (2.81 - 6.49)	4.91 (3.21 - 7.51)	2.73 (1.23 - 6.04)	2.07 (0.91 - 4.70)	3.52 (1.86 - 6.69)	2.71 (1.47 - 4.97)	1.03 (1.01 - 1.12)	1.04 (0.98 - 1.10)	1.04 (0.99 - 1.08)	0.97 (0.90 - 1.04)
CA haplotype	4.34 (2.70 - 6.97)	6.96 (4.27 - 11.33)	3.36 (1.63 - 6.94)	2.91 (1.41 - 6.04)	1.31 (0.72 - 2.36)	1.38 (0.77 - 2.46)	1.06 (1.00 - 1.11)	1.02 (0.96 - 1.08)	1.01 (0.98 - 1.04)	1.00 (0.96 - 1.03)

Haplotypes of frequencies<5% were excluded from the analysis.

12.4 μmol/l Hcy was the upper 25% cut-off value of 350 participants in survival analysis.

3.94 ng/ml FA was the lower 25% cut-off value of 350 participants in survival analysis.

Adjusted by age, gender, tumor size, tumor site, tumor differentiation, TNM stage, and chemotherapy.

Table S5

TS mRNA expression according to 3'-UTR genotypes or haplotypes

	Total (n=94)	*P*	Tumor-adjacent (n=47)	*P*	Tumor (n=47)	*P*
TS mRNA expression						
TS 1100TT	1.00±0.44 (n=36)	0.403	1.00±0.44 (n=18)	0.060	1.00±0.42 (n=18)	0.020
TS 1100TC	5.82±3.41 (n=48)		0.23±0.10 (n=24)		17.28±10.33 (n=24)	
TS 1100CC	6.03±5.77 (n=10)		0.02±0.01 (n=5)		12.21±9.36 (n=5)	
TS 1170AA	1.00±0.36 (n=42)	0.025	1.00±0.30 (n=21)	0.030	1.00±0.36 (n=21)	0.010
TS 1170AG	5.33±3.11 (n=42)		2.61±1.37 (n=21)		7.08±4.25 (n=21)	
TS 1170GG	3.67±2.15 (n=10)		5.06±3.09 (n=5)		2.15±1.08 (n=5)	
TS 1100T-1170A	1.00±0.39 (n=58)	0.024	1.00±0.48 (n=29)	0.270	1.00±0.47 (n=29)	0.008
TS 1100T-1170G	12.14±6.04 (n=62)		1.61±0.72 (n=31)		43.28±24.14 (n=31)	
TS 1100C-1170A	13.59±6.40 (n=68)		0.43±0.30 (n=34)		47.10±22.50 (n=34)	

Data were presented as mean ± standard error.

P-values were calculated by Mann-Whitney and Kruskal-Wallis tests.

Table S6

TS, miR-124-1, and miR-203 expression levels according to TS 3'-UTR polymorphisms

		Relative fold (mean±SE)		
Characteristics	N	TS	miR-124-1	miR-203
TS genotype				
1100TT	36	1.00±0.44	1.00±0.17	1.00±0.23
1100TC	48	5.82±3.41	1.08±0.18	0.94±0.26
1100CC	10	6.03±5.77	1.24±0.20	0.88±0.26
P		0.4025	0.3766	0.973
1170AA	42	1.00±0.36	1.00±0.11	1.00±0.17
1170AG	42	5.33±3.11	0.85±0.24	1.45±0.30
1170GG	10	3.67±2.15	0.48±0.22	0.39±0.28
P		0.025	0.263	0.1765
TS haplotype				
1100T-1170A	58	1.00±0.39	1.00±0.13	1.00±0.17
1100T-1170G	62	12.14±6.04	0.93±0.13	1.14±0.26
1100C-1170A	68	13.59±6.40	1.37±0.16	1.02±0.21
P		0.0238	0.159	0.876

SE, standard deviation.
P-values were calculated by Mann-Whitney and Kruskal-Wallis tests
